# The consistent difference in red fluorescence in fishes across a 15 m depth gradient is triggered by ambient brightness, not by ambient spectrum

**DOI:** 10.1186/s13104-016-1911-z

**Published:** 2016-02-17

**Authors:** Ulrike Katharina Harant, Nicolaas Karel Michiels, Nils Anthes, Melissa Grace Meadows

**Affiliations:** Animal Evolutionary Ecology, Institution for Evolution and Ecology, Department of Biology, Faculty of Science, University of Tuebingen, Auf der Morgenstelle 28, 72076 Tuebingen, Germany; Biology Department, Saint Francis University, P.O. Box 600, Loretto, PA 15940-0600 USA

**Keywords:** Phenotypic plasticity, Fluorescence, Visual ecology, Fish colouration, Chromophore, Melanophore, Tripterygiidae

## Abstract

**Background:**

Organisms adapt to fluctuations or gradients in their environment by means of genetic change or phenotypic plasticity. Consistent adaptation across small spatial scales measured in meters, however, has rarely been reported. We recently found significant variation in fluorescence brightness in six benthic marine fish species across a 15 m depth gradient. Here, we investigate whether this can be explained by phenotypic plasticity alone, using the triplefin *Tripterygion delaisi* as a model species. In two separate experiments, we measure change in red fluorescent brightness to spectral composition and ambient brightness, two central parameters of the visual environment that change rapidly with depth.

**Results:**

Changing the ambient spectra simulating light at −5 or −20 m depth generated no detectable changes in mean fluorescence brightness after 4–6 weeks. In contrast, a reduction in ambient brightness generated a significant and reversible increase in mean fluorescence, most of this within the first week. Although individuals can quickly up- and down-regulate their fluorescence around this mean value using melanosome aggregation and dispersal, we demonstrate that this range around the mean remained unaffected by either treatment.

**Conclusion:**

We show that the positive association between fluorescence and depth observed in the field can be fully explained by ambient light brightness, with no detectable additional effect of spectral composition. We propose that this change is achieved by adjusting the ratio of melanophores and fluorescent iridophores in the iris.

**Electronic supplementary material:**

The online version of this article (doi:10.1186/s13104-016-1911-z) contains supplementary material, which is available to authorized users.

## Background

Many organisms adapt to local environmental conditions in a remarkably fine-tuned way. Such adaptation typically occurs across distinct environments or habitats, often over significant spatial scales [1–4] or in situations where migration barriers restrict gene flow [5, 6]. Recent work, however, highlights that persistent adaptive differences in trait expression can also occur over comparably small spatial scales, such as a few kilometers in passerine birds [7, 8], and then usually in habitats characterized by steep environmental gradients as found along e.g. mountain slopes [9]. A recent study in marine fish, however, found persistent differences in red fluorescent color patterns at even smaller spatial scales across a depth gradient of only 15 m: fluorescence was consistently brighter in −20 m than in −5 m [10]. This depth difference coincides with a substantial shift in the spectral composition of the ambient light between the deeper stenospectral (blue–green) zone and the shallow euryspectral (full spectrum) zone [10].

**Fig. 1:**
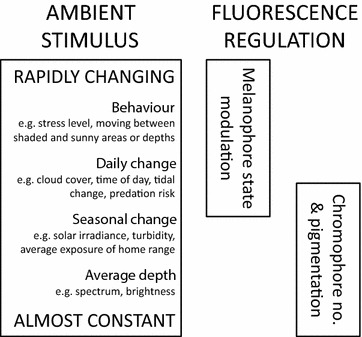
Iris fluorescence regulation mechanisms of *Tripterygion delaisi*. Melanophore state modulation is fast and covers or uncovers fluorescence as an instant response to a current chance in conditions [24]. Chromophore number and pigmentation change is much slower and is the presumed mechanism behind the relationship between depth and fluorescence [10]

Small-scale adjustments like these can be generated by genetic local adaptation or phenotypic plasticity. Phenotypic plasticity subsumes environmentally triggered plasticity within genotypes that either occurs during development and then is usually irreversible [11–13] or allows repeated and reversible fine-tuning to changing local conditions [14–16]. It remains difficult, however, to disentangle the degree to which adaptation depends on local adaptation or phenotypic plasticity [17, 18]. We test to what extent the persistent small-scale differentiation in fluorescence brightness in marine fishes can be explained by phenotypic plasticity alone, using the benthic triplefin, *Tripterygion delaisi* [19], as a model species. This species exhibits genetic sub-structure only between distinct habitats separated by kilometers of unsuitable habitat such as sand or deep water [20]. This argues against small-scale local adaptation, as is the case in other fish [21]. Hence, phenotypic flexibility seems the better explanation for short-range variation in fluorescence brightness. *T. delaisi* shows remarkably fluorescent irides, and their brightness can be down- and up-regulated within seconds [22]. The fluorescent structures in the eye of *T. delaisi*, recently described as a special type of fluorescent iridophore [22], can be uncovered or covered by an underlying layer of dendritic melanophores that regulates the fluorescent emission. However, this fast, almost instant regulatory mechanism cannot account for the persistent depth-effect found in Meadows et al. [10] (Fig. 1). Here, all fish, independent of capture depth, were held under identical light conditions for a few hours prior to and during measurement [10]. Hence, we hypothesize that fish plastically adapt the limits within which the instant regulation of fluorescence brightness shown by [22] takes place, and that they do so depending on the conditions at the depth at which they live.

Two environmental cues that are known to decrease with depth could act as stimuli for fluorescence adjustment: ambient spectrum and ambient brightness. In two separate experiments, we tested whether either of them can generate the persistent variation in fluorescence brightness that is consistent with the depth gradient in the field. In both experiments, we allowed fish collected at −20 and −5 m to adapt to controlled light conditions and assessed fluorescence brightness at regular intervals. Light conditions were then reversed to determine whether fluorescence brightness was adjusted. We predicted that fluorescence brightness increases under light conditions that represent the ambient light at depth (narrower spectrum, lower brightness). Using physiological stimulation to induce minimum and maximum fluorescence, we subsequently assessed whether the range within which fluorescence is modulated ad hoc also changed with environmental conditions, e.g. wider under depth-specific light conditions.

## Results

### Effects of collection depth

**Fig. 2:**
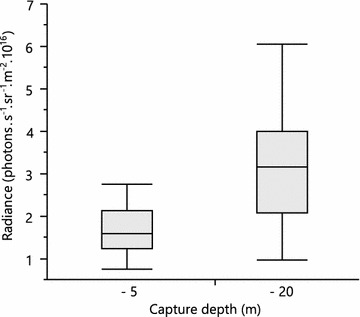
Iris fluorescence brightness at deep and shallow capture depths. Iris fluorescence brightness of *Tripterygion delaisi* measured as total photon radiance (photons s^−1^ sr^−1^ m^−2^) (*n* = 40) in the field. *Boxplots* show median (*horizontal line*), upper and lower quartiles (*boxes*) and ranges (*whiskers*)

Initially, individuals caught at −20 m (n = 20) showed significantly brighter fluorescence than individuals caught at −5 m depth (n = 20), confirming previous findings (*t* test adjusted for unequal variances, *t* = −4.5, df = 25.3, *p* < 0.001; Fig. 2). However, after exposure to a single light spectrum in the laboratory for 6 months, this depth effect disappeared (*t* = −0.5, df = 35, *p* = 0.61), confirming the existence and importance of phenotypic plasticity.

### Effects of spectral composition on fluorescence

We measured standardized fluorescence brightness in 40 fish initially exposed to euryspectral (*n* = 20) or stenospectral (*n* = 20) light spectra with identical brightness for 6 weeks and then switched each group to the alternative treatment for another 4 weeks (see “Methods”).

Contrary to our prediction, spectral composition did not affect fluorescence brightness (see Additional file 1, Fig. 3). Instead, all fish became gradually darker over the experiment, independent of treatment. This generates a pattern where fish showed brighter fluorescence under the shallow spectrum when exposed to this treatment first, but the reverse when exposed to this treatment second.

**Fig. 3:**
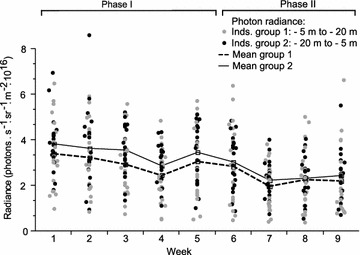
Iris fluorescence in response to spectral composition. Iris fluorescence brightness of *Tripterygion delaisi* measured as total photon radiance (photons s^−1^ sr^−1^ m^−2^) in the spectrum experiment. Fish group 1 (n = 20) started with the −20 m spectrum (phase I) and changed to the −5 m spectrum after 6 weeks (phase II), whereas group 2 (n = 20) received the opposite treatment. Fish were checked for another 4 weeks after the light switch. *Lines* represent mean total photon radiance for group 1 (*dashed*) and 2 (*solid*)

### Effects of ambient brightness on fluorescence

In the brightness experiment, fluorescence brightness was measured in fish initially exposed to ambient light of low (*n* = 9) or high (*n* = 10) overall brightness with consistent spectral composition and switched to the alternative treatment after 3 weeks (see “Methods”).

**Fig. 4:**
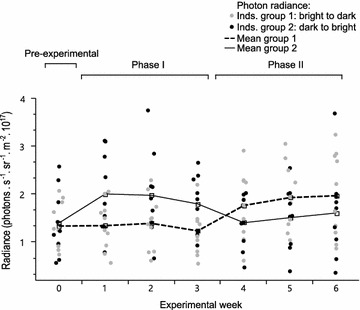
Iris fluorescence measured during the brightness experiment. Iris fluorescence of *Tripterygion delaisi* measured as total photon radiance (photons s^−1^ sr^−1^ m^−2^) throughout the brightness treatment. Group 1 (n = 10) started with the dark light treatment (phase I) and changed to the bright light treatment (phase II) after 3 weeks. Group 2 (n = 9) received the reverse treatment. *Lines* represent mean photon radiance of group 1 (*dashed*) and 2 (*solid*)

In contrast to spectral composition, ambient brightness had a highly significant effect on fluorescence brightness (Table 1; Fig. 4). Within a week, fish moved from relatively bright pre-experimental conditions into a dark light environment increased their fluorescence brightness by 43 % on average (total photon radiance from 1.4 × 10^17^ to 2.0 × 10^17^ photons s^−1^ sr^−1^ m^−2^; paired *t* test comparing initial brightness to brightness after 1 week, *t* = 5.4, df = 8, *p* < 0.001, Fig. 4), while fish moved into the bright treatment kept their initial low brightness level (1.34 × 10^17^–1.35 × 10^17^ photons s^−1^ sr^−1^ m^−2^; *t* = 0.08, df = 9, *p* = 0.93). No further change in fluorescence brightness occurred over the remaining 2 weeks the fish were kept under the same treatment (repeated measures ANOVA, bright treatment week 1–3: *F* = 0.71, df = 8, *p* = 0.12; dark treatment week 1–3: *F* = 0.2, df = 7, *p* = 0.53).

**Table 1:** Fluorescence brightness in response to ambient spectrum and brightness (adequate minimal linear mixed model)

Experiment	Parameter	Std-beta coefficient estimate	SE	*t*	*p* ^2^	R	Conditional R^2^	Marginal R^2^
Brightness/week	Intercept	17.1	0.044	384.7	<0.001			
	Brightness	0.14	0.014	9.7	<0.001		0.872	0.107
Repeatability	Bright treatment		0.034		<0.001	0.912		
	Dark treatment		0.053		<0.001	0.856		
Brightness/day	Intercept	17.13	0.061	279.61	<0.001			
	Brightness	0.246	0.056	4.34	<0.001			
	Days	0.086	0.036	2.34	0.02		0.779	0.065
Repeatability	Bright treatment		0.066		<0.001	0.791		
	Dark treatment		0.069		<0.001	0.778

The effect was reversed after switching treatments in week 4. Fish switching from bright to dark increased their fluorescence brightness significantly by 39 % within a week (1.24 × 10^17^–1.77 × 10^17^ photons s^−1^ sr^−1^ m^−2^; paired *t* test, comparing radiance measurements between week 3 and 4: *t* = 4.13, df = 9, *p* = 0.002) while fish switched from dark to bright showed a decrease of 23 % (1.8 × 10^17^–1.41 × 10^17^ photons s^−1^ sr^−1^ m^−2^; *t* = −3.83, df = 8, *p* = 0.005). In the course of the remaining 2 weeks, fluorescence brightness remained stable in the bright treatment, but tended to further increase in the dark treatment (repeated measures ANOVA: bright treatment week 4–6: *F* = 0.13, df = 7, *p* = 0.64, dark treatment week 4–6: *F* = 0.91, df = 8, *p* = 0.08).

**Fig. 5:**
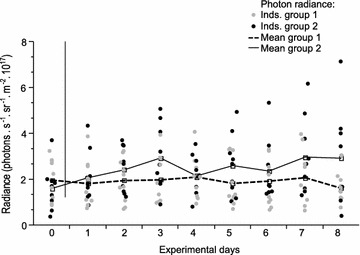
Daily change in eye fluorescence brightness measured in the last week of the brightness experiment. Iris fluorescence measured as total photon radiance (photons s^−1^ sr^−1^ m^−2^) in *Tripterygion delaisi* after the final reversal of the light conditions (*n* = 19). Experimental day 0 is identical to the measurement taken during week 6 in Fig. 5. Lights were changed in the morning of the first experimental day. Fish previously held under dark light conditions (group 1) received the bright light treatment for the following 7 days whereas fish deriving from bright light conditions (group 2) changed to the dark light treatment

In order to improve temporal resolution, we subsequently exposed fish to a second switch in light environments at the start of week 7, followed by daily measurements for 7 days. We again found a significant difference between the new treatment and the previous treatment (Table 1 brightness/day; Fig. 5). The increase in fluorescence brightness in the dark treatment occurred faster than the corresponding decrease in the bright treatment. The fish in the dark light environment already had significantly increased their fluorescence the day after the light conditions had been changed (paired *t* test = 3.032, df = 8, *p* = 0.016) and continued to increase thereafter. In contrast, fish in the bright light treatment showed a comparably small and statistically insignificant change.

### Maximum and minimum fluorescence measurements

In order to assess the instantaneous range of fluorescence that a fish can display, fish eyes were treated with either a physiological Ringer solution with elevated potassium concentration inducing melanophore contraction and maximal fluorescence exposure, or a regular Ringer solution resulting in melanophore expansion and fluorescence coverage [22]. For each individual fish, one eye underwent the maximum fluorescence treatment and the other the minimum fluorescence treatment.

**Fig. 6:**
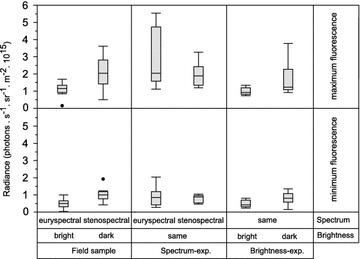
Maximum and minimum fluorescence brightness of *Tripterygion* *delaisi* eyes. Iris fluorescence brightness measured as total photon radiance (photon s^−1^ sr^−1^ m^−2^), based on 20 freshly caught fish at euryspectral (−5 m) and stenospectral (−20 m) depths, 20 fish measured after the spectrum experiment, and 19 fish measured after the brightness experiment. *Cross lines* represent significant differences between groups with significance level indicated (*p < 0.05, **p < 0.01). Note that measurements cannot be directly compared between experiments due to non-standardized measuring conditions

A second sample of fish freshly caught in the field at −20 m (n = 10) showed significantly brighter fluorescence than those collected at −5 m (n = 10; Fig. 6). This was true for the maximum fluorescence values (*t* test, *t* = −3.04, df = 12.6, *p* = 0.01) as well as for the minimum fluorescence values (*t* test, *t* = −3.63, df = 15.7, *p* = 0.002).

After the spectrum experiment, minimum and maximum values did not differ between treatments mimicking euryspectral and stenospectral conditions at −5 and −20 m depth while keeping brightness constant (*t* test minimum: *t* = −0.12, df = 18, *p* = 0.9; maximum: *t* = −0.13, df = 18, *p* = 0.89). In the brightness experiment, however, both the fluorescence maxima and minima were significantly elevated in fish kept under dark rather than bright conditions in their final treatment (*t* test maximum: *t* = 2.3, df = 17, *p* = 0.03, minimum: *t* = 2.3, df = 12.7, *p* = 0.03).

We further tested whether the total range between maximum and minimum fluorescence, and thus the range within which individuals can show instant fluorescence modulation, increased or decreased depending on the final treatment. No such difference was present in any of our treatment groups (Wilcoxon test field: *n*_euryspectral_ = 10, *n*_stenospectral_ = 10, *Z* = 0.94, *p* = 0.34; spectrum experiment: *n*_deep_ = 10, *n*_shallow_ = 10, *Z* = 1.17, *p* = 0.24; brightness experiment: *n*_bright_ = 10, *n*_dark_ = 9, *Z* = 0.37, *p* = 0.68).

## Discussion

In agreement with our general expectation, *T. delaisi* triplefins adjust their fluorescence brightness to the prevailing light conditions under a scenario of adaptive phenotypic plasticity. This response was directly triggered by the overall brightness of the ambient light environment, but was independent of its spectral composition. Our data thus support brightness-dependent changes in iris fluorescence and refute the hypothesis that previously demonstrated depth-related changes constitute a response to the stenospectral composition of light at depth.

Our observation that *T. delaisi* uses achromatic (brightness) information as an environmental trigger to adjust fluorescence brightness seems initially surprising given that brightness will vary more at a given depth with shading and daytime than spectral shape does. However, the brightness effect alone is strong enough to explain previously observed persistent differences in fluorescence brightness between shallow and deep field sites. It is not unlikely that this effect also applies to the other species for which the depth effect has been observed [10]. A new prediction following from this is that fish living in a shady part of the substrate should fluoresce more brightly than fish living at more exposed sites at the same depth.

### Subtle effects of spectrum overlooked?

Although the effect of brightness is strong enough to explain depth-related variation in fluorescence brightness, additional weak effects of spectral composition may still exist, but have gone unnoticed for two reasons. First, UV was not part of the illumination spectra. Although *T.delaisi* is unlikely to see UV (see “Methods”), their skin and iris may passively protect from UV by expressing more melanin (“tanning”) in the more UV-exposed shallow water [23]. If this effect was relevant, however, our essentially *UV*-*free* brightness treatments should not have triggered the observed significant changes in fluorescence brightness. Hence, our experiments indicate a brightness effect *independent* of UV, but a small effect of UV in the field cannot be ruled out.

Second, although differences in brightness seem to serve as the key trigger, it may still represent an adaption to local spectrum too. In the natural environment, depth, brightness, and spectral shape co-vary in a predictable way. Darker environments are more likely to be in deeper, stenospectral sites, where fish fluorescence generates stronger visual contrasts [10].

### How is fluorescence regulated?

The fact that a change in the mean fluorescence brightness coincided with a similar change in minimum and maximum values indicates that fluorescence did not just change at the instant melanophore state, but also at the tissue level. This may involve a change in iridophore optical nanostructures or in fluorescent pigment concentration. These chromatophores contain guanine/hypoxanthine crystals [24], but the identity of the fluorescent pigment contained within these crystals remains unknown. More likely, they may modify melanosome density in the melanophores or increase the number of the latter, thereby adjusting the degree to which fluorescent iridophores can be covered. Guppies, killifish, and mosquitofish disintegrate and discharge melanophores when adapting to a white background over time [25, 26]. Rice fish and goldfish even performed trans-differentiation, migration or apoptosis of their melanophores [27], similarly leading to reduced pigmentation. Little is known to date about how fast these changes occur. Nevertheless, it seems plausible that building-up melanosomes and an associated decrease in fluorescence requires more energy and time than reducing or degrading them. This could explain the temporal delay in fluorescence decrease observed in the bright light treatment during the brightness experiment compared to the relatively fast increase in the dark light treatment.

### Room for genes?

Given that phenotypic flexibility seems sufficient to explain the depth effects on fluorescence observed earlier [10], it is questionable that local genetic adaptation through natural selection is involved. However, given that there is substantial variation between individuals, our data do not exclude that a genetic component may be involved in the ability to adapt to a changing environment, irrespective of the depth at which fish are collected. This hypothesis cannot be tested using the data obtained in this study. Indirectly, our results suggest that the spatial separation between −5 and −20 m is indeed too short to result in true local adaptation for this trait.

## Conclusion

Fluorescence in *T. delaisi* is phenotypically flexible and regulated by ambient brightness, which tightly co-varies with depth and spectral shape. This finding is a major contribution to understanding the proximate reasons why fish in deeper water fluoresce more. It also offers fish at a single depth to tune their fluorescence range to prevailing light conditions determined by factors that do not change instantly, such as seasonality or degree of shading.

## Methods

### Study species, collection, and housing

*Tripterygion delaisi* is a small, cryptic, benthic triplefin from rocky habitats between −3 and −40 m depth in the Mediterranean Sea and the eastern Atlantic [28, 29]. The species possesses a prominent red fluorescent iris in which the interplay between fluorescent iridophores and covering melanophores controls fluorescence brightness [22].

We caught 100 individuals in Elba, Italy, at depths of −5 and −20 m in June 2013. Collection took place under the general permit of the Hydra Institute (Centro Marino Elba, Campo nell´ Elba, Italy). Given that this took place at the end of the breeding season, 28 males still showed breeding coloration. For most individuals, however, sex could not be inferred because both males and females display the same cryptic coloration outside the breeding season. Upon capture, fish were kept individually in perforated 1 L plastic cups placed in a 50 L flow-through tank continuously supplied with fresh seawater at ambient temperature. At the end of fieldwork and initial fluorescence measurements of all specimens (see below), fish were transferred to aquarium facilities at the Eberhard Karls University of Tübingen, Germany on 29 June 2013. They were kept individually in blue LED illuminated 15 L tanks (20 °C, salinity 34 ‰, pH 8.2, 12 h light/dark cycle, fed once per day). In order to ensure that all individuals had adapted well to the laboratory conditions and all males in breeding coloration had fully changed back to their cryptic coloration, fish were allowed to adapt to laboratory conditions for 6 months. Animal husbandry was carried out in accordance with German animal welfare legislation.

### Effects of spectral composition on fluorescence

In early December 2013, 20 fish were chosen randomly from each of the −5 and −20 m original capture depths (*n* = 40) and relocated to the experimental room into 40 individual 20 L tanks (20 °C, salinity 34 ‰, pH 8.2, 12 h light/dark cycle, fed once per day) and were allowed to adapt to their new tank for 9 days.

**Fig. 7:**
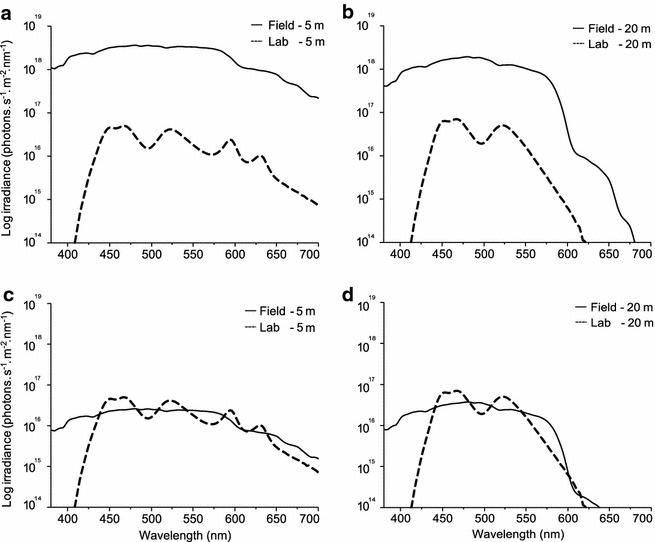
Comparison of overall brightness (**a**, **b**) and spectral shape (**c**, **d**) between field and lab measurements. Spectral curves are given as total photon irradiance (photon s^−1^ m^−2^ nm^−1^). Note that **c** and **d** represent area-normalized curves

Each aquarium was illuminated by eight LEDs in a single housing with diffusor for homogeneous lighting (custom made Feno Fe s.soft lt 18), controlled by a DMX standalone unit (Feno fc s.dmx 48d). The brightness of each LED (cold white, UV: 395–410 nm, royal blue: 450–465 nm, blue: 465–485 nm, 2× green: 520–535 nm, amber: 585–595 nm and red: 620–630 nm) could be individually controlled in 100 steps from off to maximum to generate custom spectra. We defined two light treatments to mimic the spectral shape of downwelling light in the field at −5 m (euryspectral) or −20 m (stenospectral) depth (Fig. 7). In order to assure that any effect was due to spectral shape only, care was taken to obtain identical total irradiance in the two spectral treatments (total irradiance in photons s^−1^ m^−2^, euryspectral: 2.55 × 10^18^, stenospectral: 2.51 × 10^18^). Since the light diffuser of the LED housing blocked most of the UV, the UV channel was switched off for the duration of all the experiments. Given that the eye lens of *T. delaisi* blocks UV, UV is probably irrelevant for vision (unpubl. data N. K. Michiels).

From 16 Dec 2013 onwards, half the fish of each collection depth (−5 and −20 m) received the euryspectral treatment (*n* = 10 + 10), and the other half received the stenospectral treatment (*n* = 10 + 10). Fish were kept under this illumination for 6 weeks. In order to assess changes in iris fluorescence brightness, individuals were measured at the end of each week (details below). Following this first round, the spectral treatments were reversed, exposing each individual to the alternative spectrum for another 4 weeks, again including weekly measurements. Thereafter, 20 randomly chosen fish (10 of each final treatment) were sacrificed to assess the physiological minimum and maximum fluorescence brightness (details below). One individual died during this experiment.

### Effects of ambient brightness on fluorescence

**Fig. 8:**
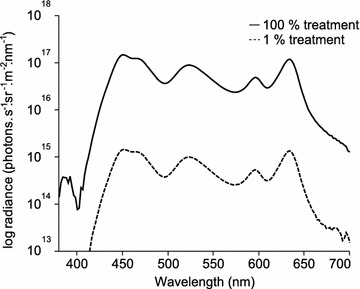
Comparison of spectral shapes between the 100 % and 1 % brightness treatment. Spectral curves are given as photon radiance (photon s^−1^ sr^−1^ m^−2^ nm^−1^)

The remaining 19 individuals, each still kept in its own tank, were kept under the same light conditions they experienced at the end of the spectrum experiment for another 11 weeks. On 19 April 2014, the fluorescence of all fish irides was measured again and fish were randomly divided into two groups. One group was exposed to 100 % white light (all light channels on 100 %) whereas the lights in the other group were completely turned off. Since spectral shapes did not affect fluorescence brightness in the spectrum experiment (see “Results”) we did not attempt to mimic the spectral shape under natural conditions in the brightness experiment, but instead used the maximum brightness possible with our light system (Fig. 8). Because bright and dark tanks were in alternating positions in the rack but separated by opaque sheets, the only light reaching the dark tanks came from diffuse reflection by the opposing white wall. As a consequence, the irradiance in the dark tanks was 1 % of that in the bright tanks (Fig. 2). This procedure was preferred over a solution with the LEDs dimmed in the dark tanks because LEDs flicker when set to lowest brightness. The tanks treated with the bright light treatment received about 70 % of the total radiance measured at a sunny day in the field at −5 m (total irradiance in photons s^−1^ m^−2^, 1.46 × 10^19^ compared to 2.05 × 10^19^), whereas the fish in the dark treatment only received about 0.8 % (total irradiance in photons s^−1^ m^−2^, 1.54 × 10^17^ compared to 2.05 × 10^19^).

As in the previous experiment, the fluorescence of the irides was measured each week for three subsequent weeks. At the start of week 4, light treatments were reversed in all tanks and measurements continued for another 3 weeks. In order to assess the daily rate of change, a final treatment reversal was performed at the start of week 7, immediately followed by a first measurement on that same day, as well as further daily measurements for another 7 days.

### Fish fluorescence measurements

All measurements were taken in a dark room. Two LED-RGB stage lights (LED Par64, 20 × 3/1 PMW, 90–240 V, 50/60 Hz) set to monochromatic blue and supplemented with a short-pass filter (ZILZ direct, Dichroic glass filter, blue) were used for fluorescence excitation in the field and spectrum experiment. In order to shorten measurement duration in individuals with weak fluorescence, which was more common in the brightness experiment, we used a brighter light source during this part of the study: blue Hartenberger Mini Compact LCD divetorch with 7 × 3.5 W 450 nm bulbs with additional short pass filter (Thorlabs FD2C subtractive dichroic color short-pass filter). In all cases, the red fluorescence of the fish iris was measured with a calibrated PR-740 SpectraScan Spectroradiometer (Photo Research Inc., bandwidth: 2 nm, aperture: 0.5, calibrated lens: MS-75, smart dark enabled, speed: normal, exposure time: automatic, extended). SpectraScan spectroradiometers have a camera-like viewfinder and lens, allowing the researcher to point it at the object of interest and to cover the area intended to be measured with a measurement spot that is adjustable in size. Because multichannel spectrometers such as the Photoresearch adjust gain to avoid saturation in the brightest wavelengths, measurements were taken through an orange filter (LEE filters, Double C.T. Orange 287) fitted over the spectroradiometer lens to suppress the blue excitation light. We determined the transmission curve of the filter and used this to calculate the original fluorescence curve of the iris. Measurements were taken at a fixed distance of 27 cm between the front edge of the lens of the spectrometer and the front of the measurement chamber. The person measuring fluorescence brightness was blind to the treatment the fish came from. Prior to the measurement, individual fish were carefully transferred into a small chamber (7 × 10 × 2.5 cm) with a black background and a thin (1.5 mm glass front) filled with seawater and placed in front of the spectrometer. Fish were positioned so that their right eye faced the glass front. The 0.5° measurement spot size covered the complete eye of *T. delaisi*. For each fish we took three measurements of the right eye and one measurement of a non-fluorescent red diffuse reflectance standard (Labsphere SCS-RD-010) to check for stray red light in the room and constancy of the measurement light conditions. The average of the red reflectance standard measurements of 16 December 2013 were used as baseline to adjust all following measurements in the spectrum experiment for variation between measurement sessions. The same procedure was used in the brightness experiment, but we used the average of the red standards from 17 April 2014 as the baseline.

Radiance was measured in W sr^−1^ m^−2^ nm^−1^. Radiance data were converted into photon radiance (photons s^−1^ sr^−1^ m^−2^ nm^−1^) and integrated to total photon radiance (photons s^−1^ sr^−1^ m^−2^) in the 525–700 nm range. The latter value was used as a measure of fluorescence brightness. Since fish reduced their fluorescence in the measurement chamber (the usual stress response to a new environment), only the highest fluorescence brightness measurement of a fish (usually the first) was used for analysis. Note that measurements are slight underestimates because 9.4 % ± 2 SD of the area measured consists of the non-fluorescent pupil.

### Maximum and minimum fluorescence measurements

Since *T. delaisi* is able to regulate its fluorescence quickly [22], we also estimated the physiological maximum and minimum fluorescence brightness an individual is able to display in its current light treatment. To this end 20 fish were sacrificed directly after the light spectrum experiment (five randomly taken from each original collection depth and final light treatment group). After the brightness experiment, all 19 remaining fish were sacrificed (10 from the bright treatment, 6 originating from −20 and 4 from −5 m; 9 fish from the dark treatment, 5 from −20 and 4 from −5 m). After decapitation, both eyes were removed. Each eye was placed on top of an eye holder (1.5 ml vial lid glued upside-down in a well of a 12-well culture plate to keep the eye facing upward). One eye was submerged in 3 ml marine physiological ringer solution (mM: NaCl 125.3, KCl 2.7, CaCl_2_ 1.8, MgCl_2_ 1.8, d-(+)-Glucose 5.6, Tris–HCl 5.0, pH 7.2) and the other in K^+^ elevated saline solution (mM: NaCl 78, KCl 50, CaCl_2_ 1.8, MgCl_2_ 1.8, d-(+)-Glucose 5.6, Tris–HCl 5.0, pH 7.2). The total ionic concentration was identical and isotonic in both. Marine physiological ringer induces melanosome dispersal in melanophores, minimising fluorescence [24]. The elevated K^+^ does the opposite, inducing melanosome aggregation and resulting in maximum fluorescence [24]. Each eye was incubated for 15 min and subsequently placed under a fluorescence microscope (Leica DM5000B) with a Leica DualCam excitation filter (480–510 nm), a Leica 550–700 nm emission filter, and a Leica EL6000 as the external light source. Measurements were taken with a c-mounted PR-740 spectroradiometer. Fluorescence brightness was calculated from radiance measurements as described above. We used a measurement spot of 0.5°, covering precisely the whole iris of the fish eye. In June 2014, the same procedure was carried out with 20 freshly caught fish (10 from −5 m and 10 from −20 m) in Calvi, Corsica, France, to provide a field reference. An overall analysis of all treated fish confirms that we obtained the envisaged effect: Elevated K+ and regular Ringer did indeed cause a highly significant gap between minimum and maximum fluorescent brightness (comparison between both eyes for all fish, paired *t* test, *t* = −8.6, df = 58, *p* < 0.001).

### Statistical analyses

General and linear mixed models were performed using the lme4 package [30] in R (R x64 3.1.1, [31]), all other analyses were performed using JMP 11 (SAS). All data were checked for normality and homoscedasticity and analysed accordingly. If possible, paired statistical tests were preferred over others to account for differences between individuals.

Backward linear mixed model selection analyses were performed for both experiments to estimate the roles of capture depth, light treatment, week (days for the final week of the brightness experiment), treatment order, the interaction between light treatment × week (days), sex, and body size (not available for the brightness experiment) on iris fluorescence. Since individual fish fluorescence radiances were measured multiple times, fish ID was included as a random factor in every step of the model selection. In the spectrum and brightness experiment, the response variable fluorescence brightness was transformed using log_10_ to approximate a normal distribution. Due to the experimental design, the time factor (week) could not be separated from a potential treatment effect in the spectrum experiment. Log_10_ iris fluorescence was therefore corrected for the week effect by using studentized residuals from a linear regression with week as predictor, irrespective of treatment or group. Model selection was then performed with the studentized residuals as a response variable. Resulting coefficient parameter estimates were standardized, allowing us to compare the factor influence between the predictors. Starting from a full model containing all fixed factors, the minimal adequate model was selected based on the Bayesian information criterion (BIC) comparing hierarchical models with and without the factor of interest. In the final model, we assessed statistical significance of each parameter using a Kenward–Roger approximation [32]. For each linear mixed model, we provide proxies for the goodness-of-fit of the fixed component (marginal R^2^) and the complete model (conditional R^2^) [33] as implemented in the piecewiseSEM package for R [34].

We estimated ANOVA-based repeatabilities for the fluorescence measurements within the three experiments (spectrum, brightness and brightness per day) as implemented in the rptR package [35] in R.

## Additional file

10.1186/s13104-016-1911-z R-scripts for all LMM´s reported in the article are provided.
