# Supplementary material for: The consistent difference in red fluorescence in fishes across a 15 m depth gradient is triggered by ambient brightness, not by ambient spectrum
Source: BMC Res Notes. 2016 Feb 17;9:107. doi: 10.1186/s13104-016-1911-z (PMC4756498; doi:10.1186/s13104-016-1911-z)
Supplement: Supplementary file 1 — 10.1186/s13104-016-1911-z R-scripts for all LMM´s reported in the article are provided. [file 13104_2016_1911_MOESM1_ESM.pdf]

Supplementary Information part I: R script used for spectrum experiment analysis

This supplementary file is organized in 3 parts. The part I is organized in 6 sections:

1. Data loading
2. Backward model selection
3. Model fit
4. Repeatability analysis
5. Graphical model evaluation
6. Results section

```
# Set paths

setwd ("I:/ulli/stat")

library (lme4) # for linear mixed model

library (ggplot2) # for graphical evaluation

library (piecewiseSEM) #package by Lefcheck 2015, function sem.model.fits(modelList) extracts marginal and condidition R² for mixed models

library (rptR) # package to establish repeatabilites

library (afex) # package to extract p-values for fixed factors
```

1.) Data loading

```
# Load data file

dat <- read.table("spectrumR.txt", header=T, sep=",", na.strings = "") # na.strings = "" transforms empty cells into real missing values = NA

str (dat)

dat$ID <- as.factor(dat$ID)

dat$Groups <- as.factor(dat$Groups)

dat$Light_treat <- as.factor(dat$Light_treat)


# Assures that column names can accessed without repeated reference to table name
```

```
attach (dat)
```

2.) Backward linear model selection

```
mod1.full <- lmer (log10_Radiance~  Light_treat + Groups + Light_treat:Groups + Capture_depth
+ Sex + Size + (1|ID), data=dat)

Summary (mod1.full)


# 1st step of model selection

mod2.a <- update (mod1.full, .~. - Light_treat:Groups)

mod2.b <- update (mod1.full, .~. - Capture_depth)

mod2.c <- update (mod1.full, .~. - Sex)

mod2.d <- update (mod1.full, .~. - Size)

# The following lines compare each of our simplified models against our current full model


anova (mod2.a,mod1.full)

anova (mod2.b,mod1.full)

anova (mod2.c,mod1.full)

anova (mod2.d,mod1.full)

# mod2d least significant / greatest decrease in BIC, so drop interaction first

mod2.full <- mod2.a


# 2nd step of model selection

mod3.a <- update (mod2.full, .~. - Size)

mod3.c <- update (mod2.full, .~. - Sex)

mod3.d <- update (mod2.full, .~. - CaptureDepth)

mod3.e <- update (mod2.full, .~. - Groups)

mod3.f <- update (mod2.full, .~. - Light_treat)
```

```
# The following lines compare each of our simplified models against our current full model
anova (mod3.a,mod2.full)
anova (mod3.c,mod2.full)
anova (mod3.d,mod2.full)
anova (mod3.e, mod2.full)
anova (mod3.f, mod2.full)
# mod2d least significant / greatest decrease in BIC, so drop Size next
mod3.full <- mod3.a

# 3rd step of model selection
mod4.a <- update (mod3.full, .~. - CaptureDepth)
mod4.b <- update (mod3.full, .~. - Sex)
mod4.c <- update (mod3.full, .~. - Groups)
mod4.d <- update (mod3.full, .~. - Light_treat)

# The following lines compare each of our simplified models against our current full model
anova (mod4.a,mod3.full)
anova (mod4.b,mod3.full)
anova (mod4.c,mod3.full)
anova (mod4.d,mod3.full)
# Light_treat can be dropped
mod4.full <-mod4.d

mod5.a <- update (mod4.full, .~. - CaptureDepth)
mod5.b <- update (mod4.full, .~. - Sex)
mod5.c <- update (mod4.full, .~. - Groups)
anova (mod5.a,mod4.full)
```

```
anova (mod5.b,mod4.full)

anova (mod5.c,mod4.full)

#Sex can be dropped

mod5.full <-mod5.b


mod6.a <- update (mod5.full, .~. - CaptureDepth)

mod6.b <- update (mod5.full, .~. - Groups)


anova(mod5.full,mod6.a)

anova(mod5.full,mod6.b)


# Groups can be dropped

mod6.full <-mod6.b


mod7.a <-update (mod6.full,.~. - Capture_depth)

anova(mod6.full,mod7.a)

#Capture_depth can be dropped as well

mod.final<- mod7.a

summary (mod.final)
```

3.) Model fit

```
# establish marginal and conditional R²

sem.model.fits(mod.final) # provides marginal R² = just fixed effects, and conditional R2 = fixed + random component


# None of the given factors could explain variation in the data set.
```

4.) Repeatability analysis

```
dat.neg <- subset(dat, treatment == “shallow")    # create data subsets within each treatment

dat.pos <- subset(dat, treatment == "deep") # create data subsets within each treatment

print(rpt.aov(dat.neg$log10_Radiance, dat.neg$ID)) #Repeatability for Gaussian Data based on ANOVA

print(rpt.aov(dat.pos$log10_Radiance, dat.pos$ID)) #Repeatability for Gaussian Data based on ANOVA
```

5.) Graphical model evaluation

```
# Divide the graphic window in 4 sub-regions

par(mfrow=c(2,2))

Err <- resid (mod.final)

Fit <- fitted (mod.final)


# A: qq plot on the residuals: normally distributed?

qqnorm (Err) # qq-plot of residuals

qqline (Err)


# B: residuals versus fitted values: no "shape"?

plot (Fit, Err)

abline (h=0, lty=2)
```

6.) Results

Df AIC BIC logLik deviance Chisq Chi Df Pr(>Chisq)

mod7.a 3 603.8059

	Df	AIC	BIC	logLik	deviance	Chisq	Df	Pr(>Chisq)
mod7.a	3	603.8059	615.7426	-298.9029	597.8059	NA	NA	NA
mod6.a	4	605.5093	621.4248	-298.7546	597.8093	0.296	1	0.586

```
# Dropping the last fixed factor capture_depth still leads to a smaller BIC therefore it can be dropped as well

# Final summary of the model without fixed effects

 summary(mod.final)

 # Output:

 Linear mixed model fit by REML ['lmerMod']
 Formula: Residual ~ (1 | ID)
    Data: dat

 REML criterion at convergence: 599.8

 Scaled residuals: 
     Min      1Q  Median      3Q     Max 
 -2.7722 -0.6107  0.1018  0.6228  2.6729 

 Random effects:
  Groups   Name        Variance Std.Dev.
  ID       (Intercept) 0.8327   0.9125  
  Residual             0.1808   0.4252  
 Number of obs: 395, groups:  ID, 40

 Fixed effects:
             Estimate Std. Error t value
 (Intercept) 0.005056   0.145878   0.035

 # Repeatability analysis output

 > print(rpt.aov(dat.deep$Residual, dat.deep$ID)) #Repeatability for Gaussian Data based on ANOVA

 Repeatability calculation using the ANOVA method

 R  = 0.829
 SE = 0.038
 CI = [0.752, 0.905]
 P  = 1.22e-49 [P.aov]
      0.001 [P.permut]

 > print(rpt.aov(dat.shallow$Residual, dat.shallow$ID)) #Repeatability for Gaussian Data based on AN
 OVA

 Repeatability calculation using the ANOVA method
```

R = 0.83
SE = 0.037
CI = [0.755, 0.905]
P = 2.06e-50 [P.aov]
     0.001 [P.permut]

R^2

> sem.model.fits(mod.final) #provides marginal R^2 = just fixed effects, and conditional R2 = fixed + random component.

	Class	Family	Link	Marginal	Conditional	AIC
1	lmerMod	gaussian	identity	0	0.8215788	603.8059

#no fixed factors included in the final model → marginal R^2 is 0

Supplementary Information part II: R script used for brightness/week experiment analysis

Part II is organized in 3 sections:

1. Data loading
2. Backward model selection
3. Model fit
4. Repeatability analysis
5. Graphical model evaluation

```
# Set paths

setwd ("D:/ulli /stat")

library (lme4)

library (ggplot2)

library (piecewiseSEM) #package by Lefcheck 2015, function sem.model.fits(modelList) extracts marginal and condidition R² for mixed models

library (rptR) # package to establish repeatabilites

library (afex) # package to extract p-values for fixed factors
```

1.) Data loading

Load data file

```
dat<-read.table("BrightnessExp_for R3.txt", header=T, sep=",", na.strings = "") # na.strings = "" transforms empty cells into real missing values = NA

str(dat)

dat$ID <- as.factor(dat$ID)

dat$Groups <- as.factor(dat$Groups)

dat$capture_depth <- as.factor(dat$capture_depth)

# Assures that column names can accessed without repeated reference to Table name
attach(dat)

mod1.full<-lmer (log10_Radiance ~  treatment + week + Groups + treatment:week + capture_depth + sex +(1|ID), data= dat)

summary(mod1.full)
```

2.) Backward model selection

```
# 1st step of model selection

mod2.a <- update (mod1.full, .~. - treatment:week)

mod2.b <- update (mod1.full, .~. - capture_depth)

mod2.c <- update (mod1.full, .~. - sex)

mod2.d <- update (mod1.full, .~. - Groups)

# The following lines compare each of our simplified models against our current full model

anova (mod2.a,mod1.full)

anova (mod2.b,mod1.full)

anova (mod2.c,mod1.full)

anova (mod2.d,mod1.full)

# mod2b least significant / greatest decrease in BIC, so drop Groups first
```

```
mod2.full <- mod2.d

# 2nd step of model selection
mod3.a <- update (mod2.full, .~. - treatment:week)
mod3.b <- update (mod2.full, .~. - capture_depth)
mod3.c <- update (mod2.full, .~. - sex)

# The following lines compare each of our simplified models against our current full model
anova (mod3.a,mod2.full)
anova (mod3.c,mod2.full)
anova (mod3.b, mod2.full)
# mod2a least significant / greatest decrease in BIC, so drop sex next
mod3.full <- mod3.c

# 3rd step of model selection
mod4.a <- update (mod3.full, .~. - treatment:week)
mod4.b <- update (mod3.full, .~. - capture_depth)

# The following lines compare each of our simplified models against our current full model
anova (mod4.a,mod3.full)
anova (mod4.b,mod3.full)
# mod4.a the least significant, drop interaction
mod4.full <- mod4.a

mod5.a <- update (mod4.full, .~. - treatment)
mod5.b <- update (mod4.full, .~. - week)
mod5.c <- update (mod4.full, .~. - capture_depth)
```

```
anova (mod4.full,mod5.a)

anova (mod4.full,mod5.b)

anova (mod4.full,mod5.c)


# capture_depth can be dropped

mod5.full <- mod5.c


# 5th step of model selection

mod6.a <- update (mod5.full, .~. - treatment)

mod6.b <- update (mod5.full, .~. - week)


# The following lines compare each of our simplified models against our current full model

anova(mod6.a,mod5.full)

anova(mod6.b,mod5.full)

# mod6.c - week - goes last

mod6.full <- mod6.b


# Check whether the model is better without the last fixed factor

mod7.a <- update (mod6.full, .~. - treatment)

anova(mod7.a,mod6.full)

# Treatment cannot be dropped! Our best model is therefore mod6.full

mod.final <- mod6.full


# For extracting p values for fixed factors

library(afex)


mixed(mod.final<-lmer(log10_Radiance ~ treatment +   (1|ID),data=dat) ,data = dat, type = 3,
method = c("KR"),
```

```
    per.parameter = TRUE, test.intercept = TRUE,
    check.contrasts = FALSE, set.data.arg = TRUE, progress = FALSE)
summary(mod.final)
```

3.) Model fit

```
# establish marginal and conditional R²
sem.model.fits(mod.final) # provides marginal R² = just fixed effects, and conditional R2 = fixed + random component
```

4.) Repeatability analysis

```
dat.neg <- subset(dat, treatment == bright")    # create data subsets within each treatment
dat.pos <- subset(dat, treatment == "dark") # create data subsets within each treatment
print(rpt.aov(dat.neg$log10_Radiance, dat.neg$ID)) #Repeatability for Gaussian Data based on ANOVA
print(rpt.aov(dat.pos$log10_Radiance, dat.pos$ID)) #Repeatability for Gaussian Data based on ANOVA
```

5.) Graphical evaluation

```
# Divide the graphic window in 4 sub-regions
par (mfrow=c(2,2))
Err <- resid (mod.final)
Fit <- fitted (mod.final)

# A: qq plot on the residuals: normally distributed?
qqnorm (Err) # qq-plot of residuals
qqline (Err)

# B: residuals versus fitted values: no "shape"?
plot (Fit, Err)
abline (h=0, lty=2)
```

Supplementary Information part III: R script used for brightness/day experiment analysis

Part III is organized in 3 sections:

1. Data loading
2. Backward model selection
3. Model fit
4. Repeatability analysis
5. Graphical model evaluation

```
# Set paths

setwd ("D:/ulli/stat")


library(lme4)

library(ggplot2)
```

1.) Data loading

```
# Load data file

dat <- read.table ("rchron3.txt", header=T, sep=",", na.strings = "") # na.strings = "" transforms empty cells into real missing values = NA

str (dat)

dat$ID <- as.factor (dat$ID)

dat$Groups <- as.factor (dat$Groups)

dat$capture_depth <- as.factor (dat$capture_depth)

View (dat)

# Assures that column names can accessed without repeated reference to Table name

attach (dat)

mod1.full<-lmer (log10_Radiance ~ treatment + Groups + week + treatment:week + capture_depth + sex + (1|ID), data = dat)

summary (mod1.full)
```

2.) Backward model selection

```
# 1st step of model selection

mod2.a <- update (mod1.full, .~. - treatment:week)

mod2.b <- update (mod1.full, .~. - capture_depth)

mod2.c <- update (mod1.full, .~. - sex)

mod2.d <- update (mod1.full, .~. - Groups)

# The following lines compare each of our simplified models against our current full model

anova (mod2.a,mod1.full)

anova (mod2.b,mod1.full)

anova (mod2.c,mod1.full)

anova (mod2.d,mod1.full)

# mod2b least significant / greatest decrease in BIC, so drop CaptureDepth first

mod2.full <- mod2.b


# 2nd step of model selection

mod3.a <- update (mod2.full, .~. - treatment:week)

mod3.c <- update (mod2.full, .~. - sex)

mod3.d <- update (mod2.full, .~. - Groups)


# The following lines compare each of our simplified models against our current full model

anova (mod3.a,mod2.full)

anova (mod3.c,mod2.full)

anova (mod3.d,mod2.full)

# mod2c least significant / greatest decrease in BIC, so drop Groups next

mod3.full <- mod3.d
```

```
mod4.a <- update (mod3.full, .~. - treatment:week)

mod4.c <- update (mod3.full, .~. - sex)


anova (mod4.a,mod3.full)

anova (mod4.c,mod3.full)

# Sex can be dropped as well


mod4.full <- mod4.c

mod5.a <- update (mod4.full, .~. - treatment:week)

anova (mod5.a,mod4.full)


# The interaction can be dropped as well


mod5.full <- mod5.a

mod6.a <- update (mod5.full, .~. - treatment)

mod6.b <- update (mod5.full, .~. - week)


anova (mod6.a,mod5.full)

anova (mod6.b,mod5.full)

# Nothing can be dropped anymore

mod.final <-mod5.full

# Test whether the model does better witout any effects

mod7.a <- update (mod.final, .~. - treatment,week)

anova (mod7.a, mod.final)

# It´s doing better with the two fixed effects

    summary (mod.final)

# For extracting p values for fixed factors use

library(afex)
```

```
mixed(mod.final<-lmer(log10_Radiance ~ treatment + week + (1|ID),data=dat) ,data = dat, type = 3, method = c("KR"), per.parameter = TRUE, test.intercept = TRUE, check.contrasts = FALSE, set.data.arg = TRUE, progress = FALSE)

summary(mod.final)
```

3.) Model fit

```
# establish marginal and conditional R²

sem.model.fits(mod.final) # provides marginal R² = just fixed effects, and conditional R2 = fixed + random component
```

4.) Repeatability analysis

```
dat.deep <- subset(dat, Light_treat == "bright")    # Create data subsets within each treatment
dat.shallow <- subset(dat, Light_treat == "dark") # Create data subsets within each treatment

print(rpt.aov(dat.bright$Residual, dat.bright$ID)) # Repeatability for Gaussian Data based on AN
OVA
print(rpt.aov(dat.dark$Residual, dat.dark$ID)) # Repeatability for Gaussian Data based on ANOV
A
```

5.) Graphical evaluation

```
# Divide the graphic window in 4 sub-regions

par (mfrow= c (2,2))    # divide the graphic window in 4 sub-regions

Err <- resid (mod.final)

Fit <- fitted (mod.final)


# A: qq plot on the residuals: normally distributed?

qqnorm (Err) # qq-plot of residuals

qqline (Err)


# B: residuals versus fitted values: no "shape"?

plot(Fit, Err)

abline(h=0, lty=2)
```
